# *Helicobacter pylori* Infection in Tripoli, North Lebanon: Assessment and Risk Factors

**DOI:** 10.3390/biology10070599

**Published:** 2021-06-28

**Authors:** Ghalia Khoder, Sara Mina, Ibrahim Mahmoud, Jibran Sualeh Muhammad, Rania Harati, Christophe Burucoa

**Affiliations:** 1 Department of Pharmaceutics and Pharmaceutical Technology, College of Pharmacy, University of Sharjah, Sharjah 27272, United Arab Emirates; 2 Sharjah Institute for Medical Research, University of Sharjah, Sharjah 27272, United Arab Emirates; rharati@sharjah.ac.ae; 3 Department of Medical Laboratory Sciences, Faculty of Health Sciences, Beirut Arab University, Beirut 11-5020, Lebanon; saramina0@gmail.com; 4 Department of Family Medicine and Behavioral Sciences, College of Medicine, University of Sharjah, Sharjah 27272, United Arab Emirates; iabdelmahmoud@sharjah.ac.ae; 5 Department of Basic Medical Sciences, College of Medicine, University of Sharjah, Sharjah 27272, United Arab Emirates; jmuhammad@sharjah.ac.ae; 6 Department of Pharmacy Practice and Pharmaceutics, College of Pharmacy, University of Sharjah, Sharjah 27272, United Arab Emirates; 7 Laboratoire de bactériologie, Hygiène, EA 4331 LITEC, CHU de Poitiers, Université de Poitiers, 86000 Poitiers, France; Christophe.BURUCOA@chu-poitiers.fr

**Keywords:** *Helicobacter pylori*, stool, Premier Platinum HpSA test, sheesha, Lebanon

## Abstract

**Simple Summary:**

*Helicobacter pylori* (*H. pylori*) has been classified as a Class I carcinogen by the International Agency of Research on Cancer (IARC) and has been identified as the most common etiologic agent of infection-associated cancers. Early detection and eradication of *H. pylori* can definitely lead to long-term healing of all *H. pylori*-related diseases. In Lebanon, the prevalence of *H. pylori* is not well documented, especially in healthy subjects. A cross-sectional study was conducted on 300 healthy Lebanese volunteers, including both children and adults. Interestingly, a significant correlation was found between *H. pylori* infection and sheesha smoking. These findings highlight the need for the development of preventive approaches and strategic indications for the appropriate management of *H. pylori* infections in Tripoli, North Lebanon.

**Abstract:**

*Helicobacter pylori* (*H. pylori*) infection occurs among half of the general population worldwide, with high geographic variability. Even though *H. pylori* is the leading cause of several gastric diseases, ranging from gastritis and peptic ulcers to gastric malignancies, such as gastric cancer and mucosa-associated lymphoid tissue lymphoma, most of the infections remain asymptomatic. Early detection and eradication of *H. pylori* can definitely prevent severe long-term gastric diseases associated with *H. pylori*. In Lebanon, the prevalence of *H. pylori* is not well documented, especially in healthy subjects. The aim of this study is to assess *H. pylori* infections and the associated risk factors in Tripoli, North Lebanon. A cross-sectional study was conducted on 300 healthy Lebanese volunteers, including both children and adults. The *H. pylori* stool antigens were detected using the Premier Platinum HpSA test. The socio-demographic data, lifestyle characteristics, and gastrointestinal characteristics of all participants were analyzed. Out of the 300 tested volunteer subjects, 31% were found to be positive for *H. pylori*. A multivariate binary logistic regression analysis for factors associated with *H. pylori* infection revealed a significant association between *H. pylori* infection and gastrointestinal disturbances, the crowding index, and occupation. A significant statistical correlation was found between sheesha smoking (*p* = 0.001) and *H. pylori* infection. These findings highlight the need for the development of preventive approaches and strategic indications for the appropriate treatment of *H. pylori* infections in Tripoli, North Lebanon.

## 1. Introduction

*Helicobacter pylori* (*H. pylori*) colonizes the stomachs of more than half of the population worldwide and is the predominant risk factor for chronic gastritis and peptic and duodenal ulcers [1]. *H. pylori* infection is associated with gastric mucosal damage, leading to chronic inflammation and gastric malignancies, especially gastric adenocarcinoma and gastric mucosa-associated lymphoid tissue (MALT) lymphoma [1,2,3]. Consequently, *H. pylori* has been identified as a Class I carcinogen and as the most common etiologic agent of infection-associated cancers by the World Health Organization (WHO) and the International Agency of Research on Cancer (IARC) [4,5]. Based on recent global statistics, gastric cancer is classified as sixth in incidence and second in mortality compared to all cancers worldwide [6]. The analysis of data from different studies shows a wide geographic variation across the world, with higher gastric cancer incidence reported in developing countries, particularly those in East Asia, Eastern Europe, and Central and South America [7]. Nevertheless, gastric cancer incidence rates have declined steadily over the last few decades due to the eradication of *H. pylori* and healthier lifestyles [8]. Despite the tremendous progress in reducing these global trends, the control of *H. pylori* infections should remain a priority. Randomized studies conducted in high-risk areas, such as East Asia, have highlighted the importance of primary and tertiary intervention in the eradication of *H. pylori* [1]. The World Health Organization encourages all countries to consider screening for *H. pylori* to reduce the outcome of gastric cancer, especially in asymptomatic populations. Several reliable tests are available to screen for *H. pylori* infections, including invasive methods, such histologic evaluation of gastric biopsies, a rapid urease test, culture, and polymerase chain reaction (PCR), as well as noninvasive methods, which include a urea breath test (UBT), IgG anti-*H. pylori* serology, and a stool antigen test (SAT) [9,10,11,12]. Most of the diagnostic tests are normally not recommended for use in epidemiological studies because they are either expensive (UBT) or they require endoscopic examination (culture, PCR) [13]. In contrast, *H. pylori* stool antigen (HpSA) tests have gained great interest in epidemiological investigations of *H. pylori* in recent years [14,15]. Several robust clinical and epidemiological studies have shown that *H. pylori* stool antigen tests using monoclonal antibodies are reliable for detecting *H. pylori* infection and are easily used, especially in children [16,17,18,19].

Transmission of the *H. pylori* infection occurs mainly through the oro-oral or feco-oral routes [20,21]. Furthermore, a high rate of transmission of the infection to sexual partners of *H. pylori*-infected subjects has been demonstrated [22]. These findings elucidated the potential risk factors associated with *H. pylori* infection. Socioeconomic conditions have been strongly related to the acquisition of *H. pylori* in developed and developing countries, particularly during childhood [23,24,25]. Other risk factors have been shown to be associated with *H. pylori* infection, such as educational level, income, living standards, such as crowding index, sanitation, and hygiene, and the source of drinking water [25].

*H. pylori* infection is mostly acquired during early childhood, increases with age, and persists throughout life in the absence of an appropriate eradication therapy [26]. The prevalence of *H. pylori* infection ranged from 22% to 87.6% in the Eastern Mediterranean region, including Saudi Arabia, Egypt, Jordan, Libya, the United Arab Emirates (UAE), Tunisia, Lebanon, and Oman [27]. A meta-analytic study on the prevalence of *H. pylori* comprising data from 62 countries documented a single study conducted in Lebanon [28]. The Lebanese prevalence study was conducted on symptomatic patients, and the prevalence was estimated to be 52% [29]. A more recent meta-analysis across 73 countries reported an overall prevalence of 44.3% and supported previous findings that showed a higher prevalence in developing countries than in developed countries [28,30].

In the last two decades, only very few *H. pylori* prevalence studies were conducted in Lebanon according to the PubMed data [29,31,32,33,34]. Only one single study investigated the prevalence of *H. pylori* in asymptomatic children in 2007 [31]. The study reported a feco-prevalence of *H. pylori* infection of 21% in 414 asymptomatic children of different socioeconomic backgrounds [31]. However, the study was performed only in children residing in Beirut, Lebanon. In the other four studies, the prevalence of *H. pylori* was mainly evaluated in symptomatic individuals with upper gastrointestinal symptoms; the prevalence was found to range from 50% to 70% [29,32,33,34]. In Lebanon, *H. pylori* infection was expected to result in half of the gastric cancer cases in 2018 [35]. However, these statistical expectations were not corroborated by recent epidemiological studies related to *H. pylori.* There have been no recent assessments of *H. pylori* infection and its risk factors in healthy Lebanese children and adults. In addition, North Lebanon and, more precisely, Tripoli have been heavily influenced by Syrian immigration in the last few years due to the proximity of this region to Syria. Due to socioeconomic problems, the majority of Syrian immigrants lived in crowded housing conditions. Therefore, including this population group in the assessment of *H. pylori* infection was one the objectives to be highlighted in the current study. In our recent epidemiological study conducted in the UAE in 2019, the prevalence of *H. pylori* was 41% [36]. Interestingly, it was found that ethnicity and profession were two of the major risks involved in increasing *H. pylori* infections [36]. In the current pilot study, we aimed to validate the risk factors previously found in the UAE on the North Lebanese population, as well as to investigate other new risk factors related to *H. pylori* infection.

The aim of this cross-sectional-based pilot study was to assess *H. pylori* infections and the risk factors in Tripoli, North Lebanon. As childhood appears to play an important role in the acquisition of *H. pylori* infection, especially in developing countries, children were included in this study in addition to adults. A noninvasive monoclonal *H. pylori* stool antigen (HpSA) test was conducted on stool samples collected from healthy children and adults. In order to determine the risk factors associated with *H. pylori* infection, the socio-demographic data, lifestyle characteristics, and gastrointestinal characteristics of the volunteer participants were surveyed. The novelty of the current study resides in that it is the first to assess *H. pylori* infections in healthy children and adults, including Syrian immigrants, as well as to determine the risk factors associated with *H. pylori* infection in the North Lebanese population. The assessment of *H. pylori* infection and its associated risk factors in North Lebanon will encourage particular public health efforts for a better treatment and prevention of *H. pylori* infection and its related gastric diseases.

## 2. Materials and Methods

### 2.1. Study Design

The cross-sectional study was carried out between May 2019 and April 2020. For an easier technical procedure, two hospitals and one governmental medical clinic located in Tripoli, North Lebanon were involved, but only as sites for stool collection. Stool samples were collected only from healthy participants who volunteered to participate in this study. Any volunteer patients from the involved hospitals and the medical clinic were strictly excluded from the study. Participants who were only residing in Lebanon, such as Syrian immigrants, were not excluded from the study. Up to 300 stool samples were collected from healthy adults and children who volunteered. A recruited research assistant was assigned to collect and freeze the stool samples daily at −20 °C. In addition, a nurse assisted participants in filling out a questionnaire and collected consent forms from all volunteer participants. In the case of the participation of children, the consent form and questionnaire were mainly filled out by the parents. To ensure understanding of the aim of the study, the questionnaire and consent forms were presented in the Arabic language for the participants. Questionnaires with ambiguity or missing answers to questions were taken out of the study. The questionnaire included three main parts. The first part contained socio-demographic and socioeconomic data, including gender, age, nationality, marital status, profession, family income, education level, number of siblings, number of rooms per house, and number of family members per room. The second part consisted of the lifestyle characteristics of the subjects. The third part comprised the family history of *H. pylori* infection and gastrointestinal disturbances. The inclusion criteria for this study were residence in Lebanon with an age of 1 year and above. The exclusion criteria included subjects treated within the two weeks previous to the study with antimicrobials, proton pump inhibitors (PPIs), or bismuth.

### 2.2. H. Pylori Stool Antigen Test

Premier Platinum HpSA (Meridian, Bioscience, Iowa City, IA, USA) was the test of choice used to detect *H. pylori* stool antigens in this study, the test consisted of a plurality of monoclonal anti-*H. pylori* antibodies adsorbed to microwells. The collected stool samples were first thawed, diluted using the dilution buffer provided in the kit, and added to each antibody-coated microwell. Positive and negative controls that were supplied within the kit were also added. According to the manufacturer’s instructions, all tests were conducted in duplicate, and a result was considered positive when absorbance was equal to or above 0.1. However, the detection of *H. pylori* in watery diarrheal stool or stool from patients under antimicrobial medications, PPIs, or bismuth preparations remains one of the main limitations of this test. For this purpose, stool collected from patients with diarrhea or under recent or ongoing antimicrobial medications, PPIs, or bismuth treatments were excluded from the study.

### 2.3. Statistical Analysis

To describe the socio-demographic data, lifestyle characteristics, and clinical characteristics of the study population, frequencies were reported with proportions. Bivariate analyses (chi-squared test) were conducted to identify the factors associated with *H. pylori* infection. A binary logistic regression model—with the primary outcome of interest being *H. pylori* infection (yes/no)—was performed using stepwise forward modeling. The omnibus tests of the model coefficients showed that the binary logistic regression models were statistically significant. Because no outliers were found and no multicollinearity was detected (variance inflation factor of <3), case-wise plots were not produced. Statistical significance was set to *p* ≤ 0.05. Several factors were included in the regression model, such as the gender, age, occupation, number of persons per room, meat consumption, sheesha smoking (Arabic hookah or tobacco pipe with a long, flexible tube connected to a water container), and gastrointestinal symptoms. The data analysis was conducted using IBM SPSS Statistics for Windows, Version 25.0 (IBM Corp., Armonk, NY, USA).

### 2.4. Ethical Statement

The Research and Ethics Committee of Beirut Arab University reviewed and approved the study [2019H-0068-HS-M-0323]. Socio-demographic data were documented with privacy. All volunteer participants signed consent forms prior to stool collection.

## 3. Results

### 3.1. Socio-Demographic, Lifestyle, and Gastrointestinal Characteristics

A total of 300 participants were enrolled in the study. The prevalence of *H. pylori* with respect to socio-demographic data, lifestyle characteristics, and gastro-intestinal characteristics is shown in Table 1. The majority of the participants were Lebanese (87%) and female (53%), with a male–female ratio of 1:1.13, and most were unemployed (80%). The ages of the participants ranged from 0 to 88 years, mostly from the age group of 30 years and above (91, 30%). The age group from 6 to 15 years constituted the smallest group of participants (29, 9.7%). The monthly household income was categorized into three groups: low (<600 USD), middle (600 to 2000 USD), and high income (>2000 USD), while the educational level was classified into four groups: no formal education and primary, secondary, and tertiary education. Forty-five percent (134, 45%) of the subjects were categorized in the low-income class, followed by 51% from the middle class and 4.3% from the class with a relatively high income. Nearly half of the participants had no formal education. More than 50% of the participants reported living in close proximity, such as three persons or above per room (228, 76%), compared to the participants living with one to two persons per room (72, 24%) (Table 1).

In terms of smoking, 20% and 21% of the participants reported smoking cigarettes or sheesha, respectively. Participants were also asked to report on direct contact with domestic animals, and the majority did not report any contact with pets.

A total of 238 (79%) participants declared that the main source of their drinking water was bottled mineral water. Regarding the source of their nutritional products, most of the participants reported higher intakes for raw vegetables (249, 83%), followed by poultry (217, 72%), meat (160, 53%), and fish (111, 37%).

Even though most of the volunteer participants were initially healthy, nearly half of the participants (52%, 157) reported experiencing at least one primary gastrointestinal disturbance, such as abdominal pain, bloating, nausea, vomiting, or burn sensations. Additionally, a family history of gastric diseases was reported by 55 participants (18%).

### 3.2. H. pylori Infection and Risk Factors

#### 3.2.1. Assessment of *H. pylori* Infection and Its Risk Factors

Ninety-four participants were positive for *H. pylori* during the study period, resulting in a prevalence of 31.33%, which included both children and adults (Table 1). The *H. pylori* infections were found more prevalent in adults above 30 years old (39%) than in children (25%), but no statistical differences were found with respect to *H. pylori* and the ages of the participants. Based on the bivariate analysis, significant differences were shown between *H. pylori* infection and several socio-demographic, lifestyle, and gastrointestinal characteristics. A significant difference in the prevalence of *H. pylori* (*p* < 0.022) was found between female participants (59, 37%) and male participants (35, 25%). In terms of occupation, the findings showed a significant relationship between *H. pylori* infection and the occupation of the participants (*p* < 0.033). Employed participants were more likely to have *H. pylori* infections (26, 43%) compared to unemployed participants or children (68, 29%). Moreover, a statistically significant difference was found between *H. pylori* infection and the number of persons per room (*p* < 0.03); participants living in a bedroom with three or more persons were more likely to be infected with *H. pylori* (30, 42%). Interestingly, *H. pylori* infection was found to be higher in sheesha smokers (*p* < 0.01), suggesting a potential role of sheesha in *H. pylori* transmission. Further studies are needed in order to highlight this finding. In addition, a statistically significant difference was found between *H. pylori* infection and gastrointestinal disturbances (*p* < 0.003). No statistical differences were found between *H. pylori* infection and the source of drinking water, nutritional resources, contact with domestic animals, the number of rooms per house, and the parents’ gastric disease history.

#### 3.2.2. Association between *H. pylori* Infection and Its Risk Factors

A multivariate binary logistic regression analysis was conducted for the following factors: gender, occupation, number of persons per room, sheesha smoking, and gastrointestinal symptoms. The results are summarized in Table 2. This table shows that among these six categorical variables, occupation (*p*  =  0.035), number of persons per room (*p*  =  0.008), sheesha smoking (*p*  =  0.001), and gastrointestinal symptoms (*p*  =  0.007) were associated with *H. pylori* infection. Employed participants had a significantly higher likelihood of being diagnosed with an *H. pylori* infection compared to unemployed participants (odds ratio (OR): 1.98, 95% CI, 1.05–3.74, *p* = 0.035). Furthermore, participants who lived in crowded conditions (≥3 members per room) were more likely to have *H. pylori* infections (OR: 2.24, 95% CI, 0.9–4.04, *p* = 0.008) compared to those who lived in a bedroom with one or two persons. In terms of sheesha smoking, it was found that participants with *H. pylori* infections used to smoke sheesha (OR: 3.81, 95% CI, 1.72–8.45, *p* = 0.001). In addition, it was found that participants with a family history of gastrointestinal problems were more prone to *H. pylori* infection (OR: 2.15, 95% CI, 1.23–3.73, *p* = 0.007) compared to those at the reference level with no family history of gastrointestinal symptoms (Table 2).

## 4. Discussion

In developing countries, *H. pylori* is a common infectious agent and a major public health problem. The current study is the first to assess *H. pylori* infection and its associated risk factors in healthy children and adults in the North Lebanese population. To date, very few studies have investigated the epidemiology of *H. pylori* in Lebanon. In the last 19 years, only five studies have investigated the prevalence of *H. pylori* in different Lebanese cities, including North Lebanon, South Lebanon, and Beirut [29,31,32,33,34]. Only one study explored the prevalence of *H. pylori* in asymptomatic children [31], while other studies have mainly assessed the prevalence of *H. pylori* in symptomatic adult patients [29,32,33,34]. Because *H. pylori* infection occurs during childhood and most of the infected subjects remain asymptomatic, screening programs for detection and prevention in the pediatric and asymptomatic population are important in order to reduce the prevalence and outcomes of the resulting gastric diseases at earlier ages.

In our study, up to 31% of the North Lebanese population that was tested was found to be infected with *H. pylori*, rendering the prevalence of *H. pylori* moderately high. It was slightly higher than that found in a similar study performed in North Lebanon twelve years ago, where the prevalence was found to be 21% among children. There are clear indications that the prevalence is still slightly increasing despite the worldwide awareness and improvement in the hygienic and sanitation conditions. The number of Syrian immigrants in North Lebanon is considered to be high due to the proximity between the two countries through the city of Tripoli. In the current study, only 13% of the tested children and adults were Syrian immigrants. However, 36% were found to be positive for *H. pylori* compared to 31% of the Lebanese subjects (Table 1). Even though the acquisition of the infection was not statistically significant, the slight difference requires further investigation and a larger-scale study in the future.

The prevalence of *H. pylori* infection in the current study was considerably low compared with findings from other studies conducted in neighboring Arab countries. In the United Arab Emirates, a similar and recent study conducted in 2019 evaluated the prevalence of *H. pylori* and found it to be 41% [36]. The assessment of *H. pylori* infections was comparable to that in a neighboring country, Turkey (25%) [37]. However, higher estimations of the prevalence (50–94%) were reported in both children and adults living in Egypt, Libya, and Saudi Arabia [27]. The highest prevalence of *H. pylori*, especially during adulthood, was shown in India and Bangladesh (90% and 88%, respectively). In an African epidemiological study, Ethiopia was reported to have the highest prevalence across the countries with 95% in the adult population [38].

According to this study, gender was not statistically associated with *H. pylori* infection in the multivariate binary logistic regression analysis. However, the bivariate analysis showed a statistical difference in *H. pylori* infections between men and women (*p* = 0.022). These findings confirmed the results of a recent global *H. pylori* prevalence study, which reported no differences in *H. pylori* infections between males and females [28]. These data provide compelling evidence of a gender disparity in *H. pylori* infections and can stimulate further research that focuses on the mechanisms that can favor the acquisition and persistence of *H. pylori*. In terms of age, our findings showed a meaningful correlation—even though it was not statistically significant—between the prevalence of *H. pylori* infection and age, where children aged from 6 to 15 years presented a higher *H. pylori* prevalence (35%) compared to younger children (less than 1 year), who presented the lowest prevalence rate (20%). This indicated that the acquisition of *H. pylori* infection can occur during the first 5 years of life, then progressively increases in prevalence with age. The high rate of *H. pylori* acquisition in older children and adults might be explained by their outdoor activities and exposure to potential external sources. These findings are in agreement with those of other studies, which showed that *H. pylori* infections progressively increase with age [39,40,41,42]. In terms of occupation, surprisingly, it was found that employed participants were found to be infected with *H. pylori* more often than unemployed participants (*p* < 0.035). This might be explained by the fact that employed participants are more exposed to outdoor activities and human communications. These findings are in disagreement with those of other studies, which state that a low socioeconomic level increases the prevalence of *H. pylori* infections [43]. In terms of living conditions, it was found that participants living in crowding conditions (≥3 members/room) were statistically more prone to the infection (*p* < 0.008). These findings were in agreement with those of other studies [38,44]. Regarding the gastrointestinal characteristics, participants suffering from gastrointestinal disturbances, such as abdominal pain, bloating, and vomiting, were significantly more exposed to *H. pylori* (*p* < 0.007). In terms of nutritional resources, no correlation was found between *H. pylori* infection and dietary habits; this is in agreement with the findings of a recent study published by Assaad et al. in 2018 [29]. In terms of smoking, no differences were found in the rates of *H. pylori* infection between cigarette smokers and non-smokers. However, a significant correlation was found between sheesha smokers and *H. pylori* infection (*p* < 0.001). This can be explained by the fact that sheesha smokers usually share the same hose while smoking. This finding can strengthen the idea of the oral transmission of *H. pylori* infections, but further investigations are needed to confirm this interesting result.

One of the most challenging parts of this study was the selection of an appropriate test for the detection of *H. pylori*. Several diagnostic tests are available on the market, but each of these tests has different purposes and limitations [9,45]. Serological tests have been widely used in epidemiological studies, but their accuracy varies from test to test [9,10]. Cultures from gastric biopsies are the most reliable test for *H. pylori* detection. However, culture tests are expensive and difficult to perform on children, and they require endoscopic examinations and exigent culture conditions. Hence, the use of this test in epidemiological studies has been restricted. Even though PCR tests are highly reliable and effective in terms of time in detecting *H. pylori*, the requirement of a gastric biopsy in order to perform PCR has rendered its usage unpractical in this prevalence study. The only test that could be used in the current study other than the HpSA was the 13C-urea breath test. The UBT has excellent performance in both adults and children, but its specificity decreases in very young children, and the collection of exhaled air is difficult in this age group. In consideration of all of the limitations of the other detection tests, Premier Platinum HpSA was adopted in this study because it is a reliable and noninvasive test with a high sensitivity and specificity [17,18,45]. One of the major advantages of the HpSA test is that it reveals active and ongoing *H. pylori* infections and does not require a phlebotomist, unlike other serological tests. The Premier Platinum HpSA test relies on the use of monoclonal antibodies that are capable of binding highly specific *H. pylori* antigens. Moreover, the test requires only small hospital laboratories with no need for advanced and specific equipment.

Moreover, the Premier Platinum HpSA test detects current *H. pylori* infections and does not require blood collection; hence, it is suitable for epidemiological studies involving children. Interestingly, the Premier Platinum HpSA test can even detect antigens of the coccoid form of *H. pylori,* which converts from the bacillary form upon exposure to antibiotic therapy [14,46]. Several studies have demonstrated that the sensitivity and specificity of *H. pylori* stool tests are comparable to those of histology, culture, or urea breath tests [14,17].

The pilot study performed here evaluated the prevalence of *H. pylori* infection and its associated risk factors in Tripoli, North Lebanon for the first time. More large-scale and multicenter epidemiological studies are needed to support these findings and to evaluate the prevalence of *H. pylori* among the Lebanese population, including the huge number of Syrian immigrants residing in Lebanon for the last five years.

## 5. Conclusions

*H. pylori* infection and its associated risk factors were assessed for the first time among healthy children and adults residing in Tripoli, North Lebanon. Up to 31% of the tested volunteer participants were found to be infected with *H. pylori*. A multivariate binary logistic regression analysis showed a significant association between *H. pylori* infection and some risk factors, such as the occupation, crowding index, and gastrointestinal disturbances of the participants. Interestingly, a significant correlation was reported for the first time between *H. pylori* infection and sheesha smoking. For prevention, it is worth taking the determined risk factors in the North Lebanese population into consideration. These findings can definitely help to support preventive and therapeutic initiatives against *H. pylori* in order to reduce the severe gastric clinical outcomes that could result from *H. pylori* infection.

## Figures and Tables

**Table 1:** Assessment of *H. pylori* infection based on socio-demographic data, lifestyle characteristics, and gastrointestinal characteristics; n (%) (*n* = 300).

Variable	Total (*n* = 300)	HP^(−)^ (*n* = 206)	HP^(+)^ (*n* = 94)	*p*-Value
**Socio-Demographic Data**				
**Gender**				
Female	159 (53)	100 (63)	59 (37)	0.022 *
Male	141 (47)	106 (75)	35 (25)	
**Nationality**				
Lebanese	261 (87)	181 (69)	80 (31)	0.510
Syrian	39 (13)	25 (64)	14 (36)	
**Age group**				
0–1	69 (23)	55 (80)	14 (20)	0.089
2–5	67(22)	49 (73)	18 (27)	
6–15	29(10)	19 (65)	10 (35)	
16–30	44(15)	26 (59)	18 (41)	
>30	91 (30)	57 (63)	34 (37)	
**Education level**				
No formal education	150 (51)	109 (73)	41 (27)	0.412
Primary	100 (34)	63 (63)	37 (37)	
Secondary	28 (9)	18 (64)	10 (36)	
Tertiary	17 (6)	12 (71)	5 (29)	
**Occupation**				
Unemployed/Children	239 (80)	171 (71)	68 (29)	0.033 *
Employed	61 (20)	35 (57)	26 (43)	
**Household Income**				
Low	134 (45)	96 (72)	38 (28)	0.367
Medium	153 (51)	103 (67)	50 (33)	
High	13 (4)	7 (54)	6 (46)	
**Number of Rooms per House**				
1	26 (9)	15 (58)	11 (42)	0.441
2	67 (22)	46 (69)	21 (31)	
≥3	207 (69)	145 (70)	62 (30)	
**Number of Persons/Room**				
1–2	228 (76)	164 (72)	64 (28)	0.030 *
≥3	72 (24)	42 (58)	30 (42)	
**Lifestyle Characteristics**				
**Cigarette Smoking Status**				
No	271 (90)	184 (68)	87 (27)	0.379
Yes	29 (20)	22 (76)	7 (24)	
**Sheesha Smoking Status**				
No	131 (79)	59 (45)	72 (55)	0.01 *
Yes	34 (21)	12 (35)	22 (65)	
**Contact with Domestic Animals**				
No	269 (90)	187 (69)	82 (31)	0.350
Yes	31 (10)	19 (61)	12 (39)	
**Source of Drinking water**				
Tap water	62 (21.)	45 (73)	17 (27)	0.456
**Mineral bottled water**	238 (79)	161 (68)	77 (32)	
**Nutritional Resources**				
Poultry	217 (72)	145 (67)	72 (33)	0.265
Raw vegetables	249 (83)	168 (67)	81 (33)	0.323
Fish	111 (37)	73 (66)	38 (34)	0.424
Meat	160 (53)	101 (63)	59 (37)	0.030
**Gastrointestinal Characteristics**				
**Gastrointestinal Disturbances**				
Absent	143 (48)	110 (77)	33 (23)	0.003 *
Present	157 (52)	96 (61)	61 (39)	
**Parents’ Gastric Disease History**				
Absent	245 (82)	169 (69)	76 (30)	0.805
Present	55 (18)	37 (97)	18 (33)	

* Significant at the level of *p* ≤ 0.05.

**Table 2:** Multivariate binary logistic regression analysis for factors associated with *H. pylori* infection (*n* = 300).

Variable	Odds Ratio (95% Confidence Interval)	*p*-Value
**Gender**		
Male	1	
Female	1.53 (0.88–2.67)	0.133
**Occupation**		
Unemployed/children	1	
Employed	1.98 (1.05–3.74)	0.035 *
**Number of persons/room**		
1–2	1	
≥3	2.24 (0.90–4.04)	0.008 *
**Raw meat**		
No	1	
Yes	1.57 (1.24–2.75)	0.115
**Sheesha smoking**		
No	1	
Yes	3.81 (1.72–8.45)	0.001 *
**Gastrointestinal symptoms**		
None	1	
Yes	2.15 (1.23–3.73)	0.007 *

* Significant at the level of *p* ≤ 0.05.

## Data Availability

Not applicable.
